# Risk Perceptions and Protective Behaviors Toward Bovine Tuberculosis Among Abattoir and Butcher Workers in Ethiopia

**DOI:** 10.3389/fvets.2018.00169

**Published:** 2018-07-24

**Authors:** Fikre Fekadu, Tariku Jibat Beyene, Ashenafi Feyisa Beyi, Bedaso Mammo Edao, Takele Beyene Tufa, Fanos Tadesse Woldemariyam, Fanta Desissa Gutema

**Affiliations:** ^1^College of Veterinary Medicine and Agriculture, Addis Ababa University, Bishoftu, Ethiopia; ^2^Centre for Outcomes Research and Epidemiology, Department of Diagnostic Medicine, Pathobiology, College of Veterinary Medicine, Kansas State University, Manhattan, KS, United States; ^3^Department of Veterinary Microbiology and Preventive Medicine, College of Veterinary Medicine, Iowa State University, Ames, IA, United States; ^4^Department of Veterinary Medicine, University of Cambridge, Cambridge, United Kingdom; ^5^Faculty of Bioscience Engineering, KU Leuven, Leuven, Belgium; ^6^Faculty of Veterinary Medicine, Ghent University, Ghent, Belgium

**Keywords:** bovine tuberculosis, health belief model, protective behavior, raw meat, risk perception, Ethiopia

## Abstract

Bovine Tuberculosis (BTB) is a serious cause of economic losses and public health threat, especially in developing countries. Humans acquire BTB through consumption of raw or undercooked meat, inhalation of aerosol and occupational exposure. A cross-disciplinary approach to study diseases connecting society and biology helps to understand the ways in which social, cultural, behavioral, and economic circumstances influence a healthy life. The objective of this study was to assess the risk perceptions and protective behaviors toward BTB among abattoir and butcher workers in central Ethiopia. A health belief model was used to generate the desired data following health belief model constructs. A total of 300 meat handlers working in local abattoirs, export abattoirs and butcher houses in Bishoftu, Modjo, Dukem, and Akaki towns of central Ethiopia were selected using a systematic random sampling method. Univariate and multivariable logistic regression analysis were used to assess factors associated with risk of exposure to BTB through the consumption of raw meat. The results showed that among the study participants, 95% heard about BTB and 93% knew that eating raw meat could be a source of BTB for humans. More than 62.7% of the respondents in the high risk group strongly agreed that contracting BTB would prevent them from coming to work, keep them in bed for an extended period of time and cause death. The majority of the respondents believed that free provision of personal protective clothing, compensation with test and slaughter campaigns, television and radio advertisements, educational programs and government-imposed penalties would help in prevention of BTB. Despite the high perceived severity and risk perception, the multivarable logistic regression model showed low-risk protective behavior among male (OR: 2.3, 95% CI: 1.2–4.3) and older age (>30) individuals (OR: 14.4 95% CI: 2.1–125.8). The study also noted the importance of media for health education as means for prevention of BTB. The authors strongly recommended the need of promotion of behavioral change toward the consumption of raw meat wich would have potential implications for the public health impacts of zoonotic tuberculosis and ultimately help national and global efforts toward prevention and control of tuberculosis.

## Introduction

Bovine Tuberculosis (BTB) is a zoonotic Tuberculosis disease (TB) caused by *Mycobacterium bovis (M. bovis)* with cattle being serving as a primary host. In 2016, an estimated 147,000 new human cases of zoonotic TB and 12,500 deaths due to the disease occurred globally. The African region carries the heaviest burden, followed by the South-East Asian region (1–3). In Africa, zoonotic TB due to *M. bovis* is transmitted through inhalation of aerosols, leading to pulmonary TB, and through ingestion of contaminated animal products such as milk and meat, leading primarily to extrapulmonary TB (4). In most developed countries, it was eliminated or controlled in the domestic animal population through strict control and eradication measures including test-and-slaughter strategies and compulsory pasteurization of milk. As a result, human infection is reduced, even though the potential risk remains in place (5, 6).

In Ethiopia, the average prevalence of bovine tuberculosis based on studies done between 2000 and 2016 showed to be 6% in cattle. The prevalence also varied based on the breeds of cattle and the production systems (7). The fact that *M. bovis* is frequently isolated from various animal organs/tissues such as lesions in the lungs and lymph nodes at slaughterhouses gestures that the disease can spread through both direct and indirect modes to human (8). Out of all human TB cases, the contribution of *M. bovis* was estimated to be 17.0% (9). This is of great importance, especially for livestock traders, farmers and animal product handlers.

Occupational exposures to *M. bovis* have been reported in many countries including Australia (10). In Nigeria, 10% prevalence of TB was diagnosed among livestock traders; and about one-quarter of the identified TB cases were caused by *M. bovis* strains. This study indicated that several factors including poor living conditions contributed to exposure of the people to *M. bovis* infections (11). In addition to the health effects, the economic loss in livestock caused by TB is enormous. Direct economic losses due to the infection become evident by 10 to 18% and 15% reduction in milk and meat production, respectively (12).

Collecting data on the status of BTB can enhance the understanding of the effects and patterns of transmission of the diseases and the associated determinant factors in population (13). To communicate the potential risks and protective measures effectively, health authorities need to understand the determinants of a particular behavior such as the role of beliefs, the perception of risk, benefits, and barriers to change to protect oneself (14, 15).

The Health Belief Model (HBM), a theory that is used to incorporate each of these factors, allows researchers to assess what might constitute one's protective behavior which is influenced by constructs of knowledge, perceived benefits, perceived susceptibility and severity, perceived barrier, self-efficacy, and cue to action (16). Addressing the occupational risks related to such infectious diseases is necessary by exploring the risk perception and protective behavior against the disease. According to this model, meat handlers at abattoirs and butcher shops are likely to overlook health-related precautionary measures including avoiding eating raw meat and refrain from contacting contaminated meat, if the meat handlers consider BTB to be a threat to their health and believe to be susceptible to the disease BTB. In other words, a meat handler and trader are less likely to eat the visibly infected parts of the meat when they feel they are at a heightened risk of BTB owing to their general work conditions such as working in the abattoirs and habits of processing raw meat with inadequate protective wear and not washing their hands before and after processing meat. A meat handler is also likely to read messages related to health if they believe that the benefits of the measures taken as a precaution to avert BTB outweigh the costs and if factors have synergistic rather than hindering contributions. The meat handlers will also need to feel that they are capable of undertaking the required actions to avoid risky behaviors which are called here self-efficacy. The cues or readiness to action component of the model is the least systematically studied or understood of all constructs (16).

However, such information is limited in Ethiopia. To this end, addressing the occupational risks related to such infectious diseases is needed by exploring the risk perception and protective behaviors against the disease. Therefore, the objective of this study was to assess the occupational exposure risk perceptions and protective behaviors toward BTB among abattoir and butcher house workers in four selected towns of central Ethiopia.

## Materials and methods

### Study site and population

**Figure 1:**
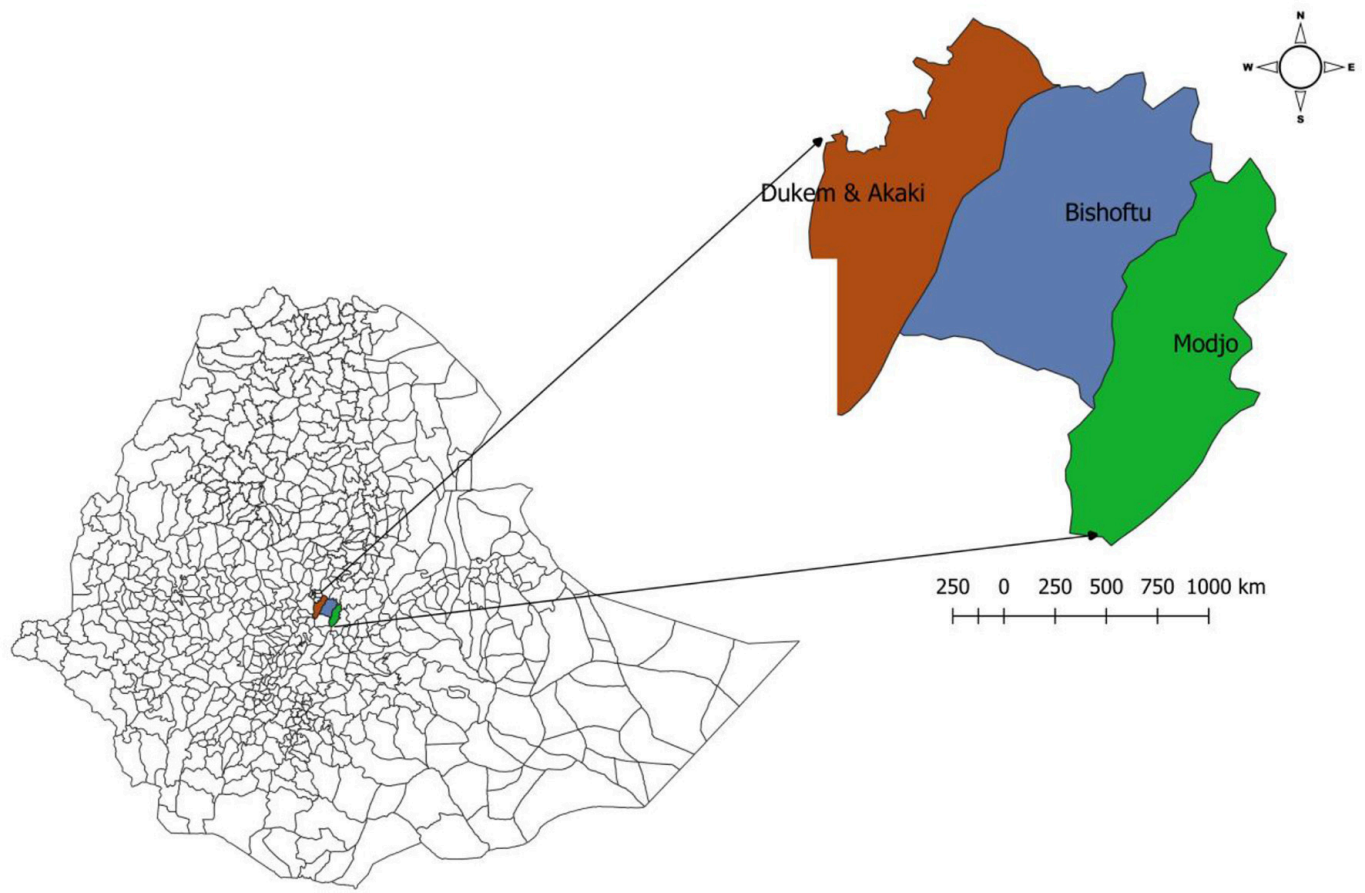
Map of the study sites.

The study was conducted in four selected towns in central Ethiopia (Bishoftu, Modjo, Dukem, and Akaki towns) on randomly selected people working in local and export abattoirs as well as butcher houses (Figure 1). The study population consists of people living in the towns working in abattoirs and butcher houses. The eligibility criteria were meat handlers who were 15 years old or above, working in local or export abattoirs and butcher houses.

### Study design and theoretical framework

The study employed a cross-sectional study design following the HBM. The model is commonly used to explain a wide variety of health behaviors and can be successfully used to guide public health interventions (16). It emphasizes the subjective perceptions of the individuals in understanding behaviors. Perceived susceptibility and severity of a health hazard as well as perceived benefits and barriers of preventive health behaviors are key components of the HBM. They are theorized to underline the cognitive processes involved in health-related decision making (17). The current study followed similar protocol used by Hambolu et al. (18) in adopting the HBM.

The main study outcome was whether respondents did or did not eat raw meat. Those who ate raw meat were classified as ‘'high risk” and otherwise “low risk.” The independent variables were related to socio-demographic variables, knowledge indicators related to TB and BTB, other risky behaviors related to BTB, participants' perceived susceptibility, perceived severity, perceived barriers, self-efficacy, and cues to action to BTB.

### Sample size and sampling

The sample size was calculated by considering 74.4% expected prevalence of raw meat eating habit (16), 95% confidence interval and 5% required precision. Accordingly, the minimum target sample size was 289 and we collected data from 300 study people working the abattoirs in the study area.

### Data collection tool and eligibility

Data were collected through face-to-face interview using a pre-tested and structured questionnaire. The questionnaire format consisted of four sections. The first part included questions about participants' socio-demographic characteristics such as age, gender, level of education, monthly income, and religion. The second part comprised of questions examining the knowledge on BTB, with response options of “yes,” “no” or “I don't know.” The third part had items asking about risk-taking behavior, including whether participants eating raw meat with response options of either “yes” or “no.” The final part consists of questions relating to each of the health belief model constructs: perceived susceptibility, severity, barriers, self-efficacy, and readiness or cues to action. For the items in the health belief model constructs, participants were asked to indicate their level of agreement to the given statements eliciting their own views on a five-point Likert scale:(1) Strongly disagree, (2) Disagree, (3) Neither agree nor disagree, (4) Agree, and (5) Strongly Agree.

### Statistical analysis

The collected data were entered into excel spreadsheet and analyzed using SPSS version 20. As all the variables were categorical, the values in each category were presented together with their corresponding percentages. Univariate and multivariate logistic regression analyses were conducted to examine the effects of the independent variables on the dependent variable (eating raw meat). Correlation between responses to items within a construct was tested using Cronbach alpha. If the correlation was high (> 0.7), then the average of the Likert scale was considered. Candidate variables having a *P*-value less than 0.05 during the univariate analysis were further included in the multivariate logistic regression model to see their association with risk of consuming raw meat. The significance level was set at α <0.05 (19).

## Results

### Socio-demographic characteristics

A total of 300 people were interviewed and all responded to the questionnaires. Of these, 75% (225) of the respondents had eaten/consumed raw meat and categorized as having high-risk behavior whereas the remaining who did not consume raw meat (25%) were referred as “the low-risk group.”

Eighty percent (241/300) of the respondents were male, and more than half of them (165/300) were in the age category of 21–30 years. The univariate analysis showed that among the socio-demographic variables, only the gender variable was shown to have a significant difference across proportions of categories of a variable as compared between low and high risk groups (*P* < 0.01). Irrespective of the risk category, people in the categories of male gender, 21–30 age range, Orthodox religion followers, those who work in abattoirs, and those with income range of 1–5 USD per day where found to make 80.3, 55, 83.3, 69.3, and 97% of the participants having the habit of consuming raw meat, respectively (Table 1).

**Table 1:** Univariate analysis of demographic characteristics and their association with raw meat eating habit of abattoir and butcher house workers.

**Variables**	**Number (%)** ***n* = 300**	**High risk (%)** ***n* = 225**	**Low risk (%)** ***n* = 75**	***p*-value**
**GENDER**				
Male	241(80.3)	153 (68.0)	63(84.4)	<0.01
Female	59 (19.7)	72(32.0)	12(15.6)	
**AGE**				
15–20	18 (6.0)	21(9.3)	4(4.9)	0.06
21–30	165 (55.0)	135(60.0)	40(53.3)	
31–40	68 (22.7)	42(18.7)	18(24.1)	
>40	49 (14.3)	27(12.0)	13(17.7)	
**LEVEL OF EDUCATION**				
Illiterate	11 (3.7)	12(5.4)	4(4.9)	0.66
At school	274 (91.3)	200(89.3)	69(92.0)	
Graduate	15 (5.0)	12(5.3)	2(3.1)	
**RELIGION**				
Orthodox	250 (83.3)	198(88.0)	61(81.8)	0.46
Muslim	18 (6.0)	12(5.3)	5(6.2)	
Protestant	27 (9.0)	15(6.7)	7(9.8)	
Traditional	5 (1.7)	0(0.0)	2(2.2)	
**OCCUPATION**				
Abattoir worker	208 (69.3)	171 (76.0)	50 (67.1)	0.19
Butcher man	92 (30.7)	54 (24.0)	25 (32.9)	
**INCOME PER DAY**				
<1 USD	6 (2.0)	3 (1.3)	2 (2.2)	0.85
1–5 USD	291 (97.0)	219 (97.3)	73 (96.9)	
>6 USD	3 (1.0)	3 (1.3)	1 (0.9)	

### Knowledge

Among the respondents, about 95.3% of the respondents had awareness about TB. In spite of this fact, 97.3% of them were found to consume raw meat becoming a high-risk group. Ninety-three percent of all the interviewed people knew about the transmission mode of TB from animals to humans. More than 70% of them were aware about that the healthy-looking meat could be contaminated. On the other hand, about 90% of respondents knew that consumption of contaminated meat could be a source of BTB in humans (Table 2). Based on the univariate analysis, all the variables related to knowledge about BTB: heard of TB, spread of TB from animals to humans, healthy-looking meat contains TB causing pathogen and consumption of contaminated meat can be a source of infection in humans were found statistically associated with the high-risk behavior of the habit of consuming raw meat (*P* < 0.05).

**Table 2:** Univariate analysis of knowledge about bovine tuberculosis (BTB) by risk categories among workers of abattoirs and butcher shops.

**Knowledge related variables**	**Number (%)**	**High risk (%)** ***N* = 225**	**Low risk (%)** ***N* = 75**	***p*-value**
**HAVE YOU HEARD OF TB?**				
Yes	286 (95.3)	201 (89.3)	73 (97.3)	0.02
No	2 (0.7)	3 (1.3)	3 (0.4)	
Don't Know	12 (4.0)	33 (14.7)	33 (3.6)	
**CAN TB SPREAD FROM ANIMALS TO HUMANS?**				
Yes	279 (93.0)	192 (85.3)	72 (95.6)	<0.01
No	2 (3.3)	0 (0)	1 (0.9)	
Don't know	19 (4.0)	33 (14.7)	3 (3.6)	
**CAN HEALTHY LOOKING MEAT CONTAIN TB CAUSING PATHOGENS?**				
Yes	221 (73.7)	138 (61.3)	58 (77.8)	0.02
No	25 (8.3)	24 (10.7)	6 (7.6)	
Don't know	54 (18.0)	63 (28)	11 (14.7)	
**IS CONSUMPTION OF CONTAMINATED MEAT A SOURCE OF BTB INFECTION IN HUMANS?**				
Yes	267 (89.0)	186 (82.7)	68 (91.1)	<0.01
No	6 (2.0)	0 (0)	2 (2.7)	
Don't know	27 (9.00)	39 (17.3)	5 (6.2)	

### Perceived susceptibility

The univarate logistic regression analysis showed statistically significant association of all the considered evidence for the respondents' perceived susceptibility with the high risk behavior for contracting BTB (*P* < 0.05). Most of respondents perceived that they had a probability of increased chance of contracting BTB because of their work, when they use bare hands, when they would eat in the slaughterhouses and perceived that contaminated (unwashed) hands and eating raw meat (Table 3).

**Table 3:** Univariate analysis of perceived susceptibility to bovine tuberculosis (BTB) by risk groups (Percent sum up to 100 for each risk group across the levels of Likert scales.

**Questions**	**Strongly disagree (%)**	**Disagree (%)**	**Neither agree nor disagree (%)**	**Agree (%)**	**Strongly agree (%)**	***p*-value**
**DO YOU THINK THAT YOU HAVE AN INCREASED CHANCE OF CONTRACTING BTB BECAUSE OF YOUR WORK?**						
Low risk	26.7	2.7	6.7	30.7	33.3	0.02
High risk	19.1	2.2	2.2	22.2	53.3	
**DO YOU THINK THAT YOU ARE AT INCREASED RISK OF CONTRACTING BTB WHEN YOU USE A BARE HAND?**						
Low risk	26.7	1.3	6.7	36.0	29.3	<0.01
High risk	17.3	1.3	2.2	27.1	52.0	
**DO YOU THINK THAT YOU ARE AT INCREASED RISK OF CONTRACTING BTB WHEN YOU EAT IN THE SLAUGHTER SLAB?**						
Low risk	24.0	1.3	6.7	41.3	26.7	<0.01
High risk	10.7	0.4	8.5	27.2	52.7	
**DO YOU THINK THAT YOU ARE AT INCREASED RISK OF CONTRACTING BTB WHEN YOU DON'T WASH YOUR HANDS AFTER HANDLING CARCASSES?**						
Low risk	24.3	1.4	4.1	36.5	33.8	0.01
High risk	14.2	0.4	2.7	25.2	57.1	
**DO YOU THINK THAT YOU ARE AT INCREASED RISK OF CONTRACTING BTB WHEN YOU EAT RAW MEAT?**						
Low risk	69.3	1.3	4.0	14.7	10.7	<0.01
High risk	15.5	0.9	2.7	45.1	35.4	

### Perceived barriers to prevention

Contrary to the perceived susceptibility all the attributes of the perceived barriers to prevention of BTB were not statistically associated with the high-risk behavior (*P* > 0.05) (Table 4).

**Table 4:** Univariate analysis of perceived barriers to prevent bovine tuberculosis (BTB) among workers of abattoirs and butcher shops.

**Questions**	**Strongly disagree (%)**	**Disagree (%)**	**Neither agree nor disagree (%)**	**Agree (%)**	**Strongly agree (%)**	***p*-value**
**DO YOU NEED TO TASTE MEAT BEFORE SELLING TO SHOW THAT IT IS SAFE?**						
Low risk	80.0	0.0	0.0	1.3	18.7	0.36
High risk	72.0	0.0	0.0	0.9	26.8	
**CANNOT WEAR PROTECTIVE CLOTHING BECAUSE THEY ARE NOT CONDUCIVE TO WORK?**						
Low risk	94.7	0.0	0.0	4.0	1.3	0.11
High risk	98.2	0.9	0.9	0.4	0.9	
**CANNOT WEAR PROTECTIVE CLOTHING BECAUSE THEY ARE EXPENSIVE?**						
Low risk	98.7	0.0	0.0	1.3	0.0	0.30
High risk	99.6	0.4	0.0	0.0	0.4	
**DO NOT WEAR PROTECTIVE CLOTHING BECAUSE MY COLLEAGUES DO NOT?**						
Low risk	98.7	0.0	0.0	1.3	0.0	0.26
High risk	99.1	0.4	0.0	0.0	0.9	

### Perceived severity

More than 62.7% of the respondents in the high risk group strongly agreed that contracting BTB would prevent them from coming to work, keep them in bed for an extended period of time and cause death. These were statistically significant (*p* < 0.05). There were no significant difference between high risk and low-risk groups based on contacting BTB is scaring and treatable or not (*P* > 0.05) (Table 5).

**Table 5:** Univariate analysis of perceived severity to prevent bovine tuberculosis (BTB) among workers of abattoirs and butcher shops.

**Questions**	**Strongly disagree**	**Disagree**	**Neither agree nor disagree**	**Agree**	**Strongly agree**	***P*-value**
**DO YOU THINK THAT CONTRACTING BTB WILL PREVENT YOU COMING TO WORK?**						
Low risk	6.7	0.0	5.3	29.3	57.3	<0.01
High risk	2.7	0.0	1.3	17.3	78.7	
**DO YOU THINK THAT CONTRACTING BTB WILL KEEP YOU IN BED FOR AN EXTENDED PERIOD OF TIME?**						
Low risk	10.7	0.0	4.0	28.0	56.0	0.02
High risk	4.4	0.0	1.3	20.0	74.2	
**DO YOU THINK THAT CONTRACTING BTB SCARES YOU?**						
Low risk	29.3	4.0	4.0	14.7	46.7	0.12
High risk	21.7	1.8	1.3	12.4	62.7	
**DO YOU THINK THAT BTB CAN CAUSE DEATH**						
Low risk	5.3	2.7	2.7	22.7	65.3	0.01
High risk	2.7	0.4	1.8	10.7	84.4	
**DO YOU THINK THAT TB IS TREATABLE?**						
Low risk	5.33	2.7	4.0	12.0	74.7	0.53
High risk	3.6	0.9	2.2	10.2	83.1	

### Self-efficacy

Only 25% of the respondents in the high risk group agreed or strongly agreed that they were able to tell if carcasses were infected with TB or not (*P* < 0.05). There were no significant difference between high risk and low-risk groups based on the capacity to buy protective wear and wearing of protective wear when their colleagues are not wearing *P* > 0.05 (Table 6).

**Table 6:** Univariate analysis of self- efficacy to prevent bovine tuberculosis (BTB) among workers of abattoirs and butcher shops in Central Ethiopia.

**Questions**	**Strongly disagree**	**Disagree**	**Neither agree nor disagree**	**Agree**	**Strongly agree**	***P*-value**
**CAN YOU BUY PROTECTIVE WEAR?**						
Low risk	85.3	2.7	1.3	1.3	9.3	0.25
High risk	78.7	0.9	0.4	4.4	15.6	
**CAN YOU WEAR PROTECTIVE WEAR EVEN IF YOUR COLLEAGUES ARE NOT?**						
Low risk	1.3	0.0	1.3	1.3	96.0	0.39
High risk	1.3	0.0	0.0	1.3	97.3	
**ARE YOU ABLE TO TELL IF CARCASSES ARE INFECTED WITH TB OR NOT?**						
Low risk	76.0	6.7	2.7	2.7	12.0	<0.01
High risk	63.1	2.2	0.9	25.3	8.4	

### Cue to action

Over 65% of respondents in the high risk group agreed or strongly agreed that free provision of protective clothing and compensation with test and slaughter campaigns would help to protect BTB (*P* < 0.05). Even though, not statically significant (*P* > 0.05), the majority of the respondents agreed or strongly agreed that both television and radio advertisements, educational programs, and government-imposed penalties would help to protect BTB (Table 7).

**Table 7:** Univariate analysis of Cues to action to prevent bovine tuberculosis (BTB) among workers of abattoirs and butcher shops in Central Ethiopia.

**Questions**	**Strongly disagree**	**Disagree**	**Neither agree nor disagree**	**Agree**	**Strongly agree**	***P*-value**
**DO YOU THINK THAT EDUCATIONAL PROGRAMS WOULD HELP TO PROTECT BTB?**						
Low risk	5.3		5.3	32.0	56.0	0.29
High risk	4.4		2.2	26.2	67.1	
**DO YOU THINK THAT SUPPLY OF FREE CLOTHING WOULD HELP TO PROTECT BTB?**						
Low risk	4.0	0.0	9.3	34.6	52.0	0.01
High risk	1.3	0.4	2.2	27.1	68.8	
**DO YOU THINK THAT ADEQUATE COMPENSATION FOR COOPERATING WITH TEST AND SLAUGHTER CAMPAIGNS WOULD HELP TO PROTECT BTB?**						
Low risk	5.3		13.3	34.6	44.0	<0.01
High risk	1.7		4. %	28.4	65.3	
**DO YOU THINK THAT GOVERNMENT IMPOSED PENALTIES WOULD HELP TO PROTECT BTB?**						
Low risk	2.6	2.6	6.6	22.6	64.0	0.06
High risk	2.2	0.0	2.6	25.3	69.7	
**DO YOU THINK THAT TELEVISION ADVERTISEMENT WOULD HELP TO PROTECT BTB?**						
Low risk	1.3		5.3	20.0	72.0	0.66
High risk	0.8		2.6	18.6	77.7	
**DO YOU THINK THAT RADIO ADVERTISEMENT WOULD HELP TO PROTECT BTB?**						
Low risk	1.3		5.3	18.6	73.3	0.39
High risk	0.8		1.7	18.6	78.6	

**Figure 2:**
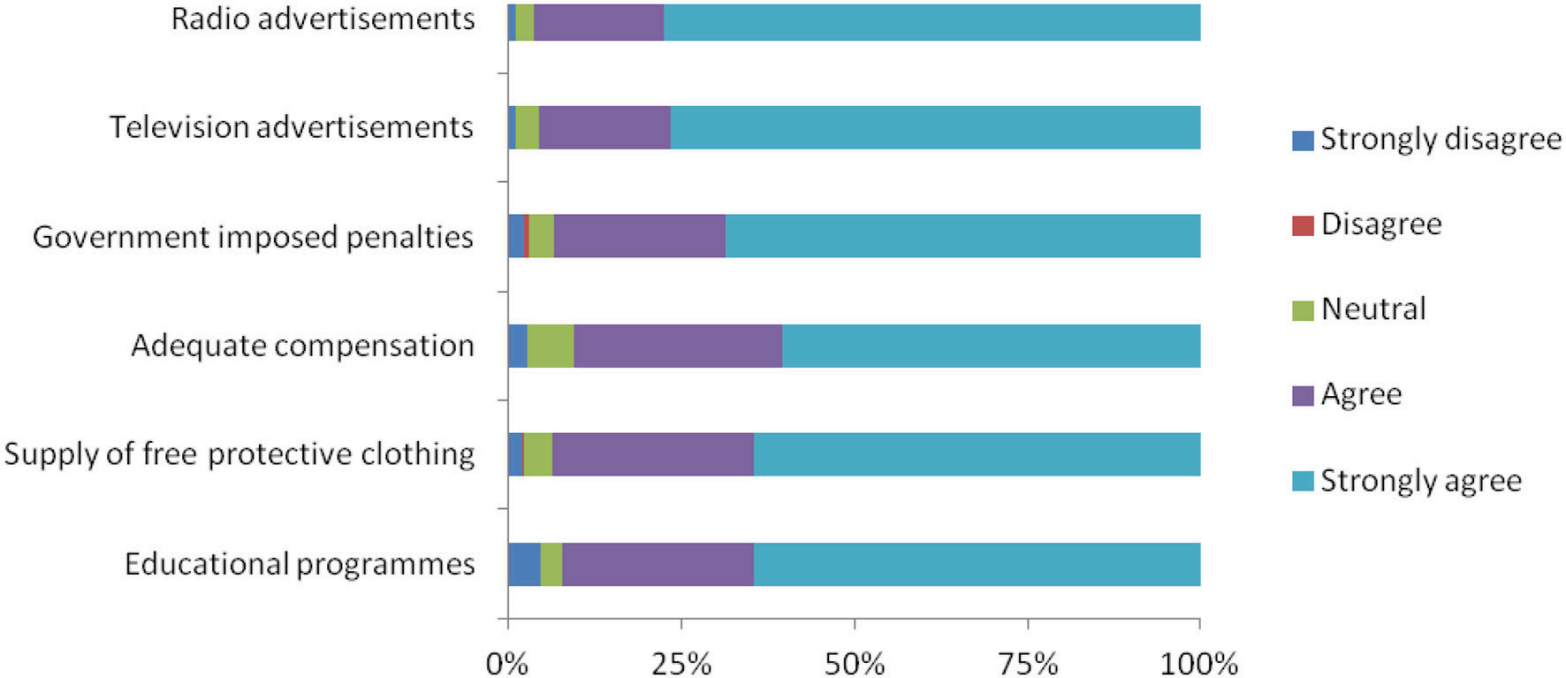
Major interventions for facilitating the adoption of protective behaviors and practices.

Evaluation of the way the public protection could be prompted shows that the majority of respondents agreed or strongly agreed that radio advertisements and adequate compensation would help (Figure 2). In addition, about 65% of respondents felt that they would need educational programs and free provision of protective clothing in order to comply with the procedures and 60% felt that government-imposed penalties for those who do not practice safe measures would work.

All the potential predictors of the high-risk behavior for contacting BTB under each construct of HBM based the univariate analysis having statistically significant association at a *p*-value less than 0.05 and high correlation (>0.7) between significant variables with in each construct were further analyzed using multivariate logistic regression to determine the predictors of the high risk behavior. The analyses were done using the significant variables and averaged Likert scale of the significant variables under each construct. Accordingly, only the male gender, those who claim to be older and those who perceive that are not susceptible to BTB were associated with the risk of high risk behavior of consuming raw meat (Table 8).

**Table 8:** Multivariate logistic regression analysis of HBM constructs to prevent bovine tuberculosis (BTB) among workers of abattoirs and butcher shops.

**Risk factors identified**	**Adjusted odds ratio (95% CI)**	***P*-value**
**PERCEIVED SUSCEPTIBILITY**		
High susceptibility	1	
Low susceptibility	1.6 (1.2–2.1)	<0.01
**GENDER**		
Female	1	
Male	2.3 (1.2–4.3)	0.01
**AGE CATEGORY**		
<15	1	
15–20	4.6 (0.6–44.2)	0.15
21–30	6.4 (0.9–51.5)	0.15
31–40	14.4 (2.1–125.8)	<0.01
>40	9.6 (1.3–89.9)	0.03

## Discussion

This study attempted to assess the risk perceptions and protective behaviors on BTB and identify the determinants of the high-risk behavior, eating raw meat, among workers of abattoirs and butcher shops in central Ethiopia using the health belief model. The present study showed the high prevalence of the risky behavior of eating raw meat for BTB (75%, 225/300), which was in agreement with the findings of Biru et al. (20) in which 79.3% people were found to consume raw meat in and around Sululta, central Ethiopia. About 95% of the respondents were aware of TB, and 93% of them were aware that TB can spread from animals to humans and the was relatively higher as compared to a previous study (21), which reported that about 82% of the respondents in western highland regions of Cameroon aware of TB. The high-risk group was found to exhibit better knowledge (95.3%) about TB, despite that, they were found to consume raw meat becoming a high-risk group. This is not in line with theories of the health belief model as well as other health behavior models, which might be due to the longtime and deep entrenched cultural habit of eating raw meat in Ethiopia, particularly eating “*kurt*” (raw beef) and “*kitfo*” (raw or undercooked minced beef mixed with blend of several spices) in many social groups including educated people such as animal and human health professionals in the country (22).

The health belief model recognizes the importance of raising awareness in the populations for the promotion of health and disease protective life strategy. Our finding was in contrary to other findings that concluded as “patchy awareness” and lack of knowledge of zoonosis combined with raw meat eating habits and poor livestock keeping systems are likely to expose respondents to an increased risk of contracting zoonosis (18, 23).

Out of the demographic factors male gender and age (above30 years) were found to associate with the high-risk behavior, consumption of raw meat. This might be due to the fact that most of the workers in the abattoir were male individuals (80.3%). The finding of risky behavior related to the age was not in agreement with another finding, this might be due to the raw meat eating culture of adult people as compared to young ones in Ethiopia (22). In this study, the male respondents were found to be more in a high-risk group compared to the female counterpart. This finding is in agreement with the reports of Hambolu et al. (18) who reported 78.2% of males were in the high group in Nigeria. As the matter of the fact and the high probability of the exposure, older groups and male individuals working in abattoirs in Ethiopia will be at greater risk of contracting BTB. Behavioral sciences explain that the observed predominance of risk-bearing behavior among males as inherently linked to the social construction of masculinity in many African countries. Given that, further in depth studies might be required to get insight into the Ethiopian context (23).

In our study, even though they were not statistically significant, free provision of personal protective clothing, compensation with test and slaughter campaigns wherever economic benefits allow, television and radio advertisements, educational programs and government-imposed penalties found to help to protect BTB. These findings are comparable with a study conducted in Nigeria (18).

The respondents perceived susceptibility to contracting BTB showed that there was increased chance of contracting BTB because of handling meat using a bare and contaminated hand, their work, eating at slaughter slab and eating raw meat. In terms of perceived barrier namely the perception that one cannot wear personal protective clothing because they are not conducive for work, and the perception that one cannot sell meat without tasting were not found to be predictors of the high-risk behavior. However, according to Janz et al. (24) the perceived barriers were the most important predictors of behavior while perceived susceptibility was the most important amongst predictors of preventative behavior.

The main limitation of this study was the use of cross-sectional study design, which is unable to verify causal relationships between the dependent and independent variables. It is documented that other methods such as longitudinal designs have a clear superiority in studies of belief-behavior relationships (25). The face to face semi-structured interviews which were used in this study might have increased the likelihood of respondents' inclination to give socially acceptable answers as also hypothesized by Hambolu et al. (18). Despite the limitation, there was a high response rate (100%), making the results likely to be the beliefs of the study population.

In conclusion, the study revealed low-risk protective behavior among male and older age (>30) individuals despite the high-risk perception and the importance of media for health education as means for prevention of BTB. We believe that the findings of the study would help and serve as a baseline data for policy and decision makers to take appropriate actions aimed at mitigating the risk of tuberculosis transmission to humans from animals following consumption of raw meat. Avoiding eating raw meat, avoiding handling of meat using bare and contaminated hands, creation of awareness for workers in abattoirs and butcher houses in particular and the general population in general about zoonotic importance of BTB using radio and television streaming and a national level study to assess the public perception regarding zoonotic importance of BTB were recommended.

## Ethics approval and consent to participate

This research was approved by the Academic Commission of College of Veterinary Medicine and Agriculture, Addis Ababa University. As the research was not involving invasive procedures and collection of biological samples, separate ethical clearance was not solicited. The study purpose was explained to participants and verbal agreement was obtained before proceeding to the study. Due to the low literacy rate and lack of interest of the participants to put their signature on consent form, the researchers opt to collect the desired data merely based on oral consent.

## Author contributions

FF collected field data. TB, FG, AB, FW conceived the idea of the study, participated in the study and questionnaire design, organized the data collection, wrote the draft of the manuscript, and completed the final version for submission. TB analyzed the data and interpreted the results. TT participated in developing the research idea and study design. BE participated in the development of the idea, supervised the work, and edited the final version of the manuscript before submission.

### Conflict of interest statement

The authors declare that the research was conducted in the absence of any commercial or financial relationships that could be construed as a potential conflict of interest.
